# Public attitudes toward cloud computing and willingness to share personal health records (PHRs) and genome data for health care research in Japan

**DOI:** 10.1038/s41439-023-00240-1

**Published:** 2023-03-30

**Authors:** Mayumi Kusunose, Kaori Muto

**Affiliations:** 1 The Graduate School of Frontier Sciences, The University of Tokyo, Tokyo, Japan; 2 The Center for Integrative Medical Sciences, RIKEN, Yokohama, Japan; 3 The Department of Public Policy, The Institute of Medical Sciences, The University of Tokyo, Tokyo, Japan

**Keywords:** Ethics, Health policy, Medical ethics

## Abstract

Japan’s government aims to promote the linkage of medical records, including medical genomic testing data and personal health records (PHRs), via cloud computing (the cloud). However, linking national medical records and using them for health care research can be controversial. Additionally, many ethical issues with using cloud networks with health care and genome data have been noted. However, no research has yet explored the Japanese public’s opinions about their PHRs, including genome data, being shared for health care research or the use of the cloud for storing and analyzing such data. Therefore, we conducted a survey in March 2021 to clarify the public’s attitudes toward sharing their PHRs, including genome data and using the cloud for health care research. We analyzed data to experimentally create digital health basic literacy scores (BLSs). Our results showed that the Japanese public had concerns about data sharing that overlapped with structural cloud computing issues. The effect of incentives on changes in participants’ willingness to share data (WTSD) was limited. Instead, there could be a correlation between WTSD and BLSs. Finally, we argue that it is vital to consider not only researchers but also research participants as value cocreators in health care research conducted through the cloud to overcome both parties’ vulnerability.

## Introduction

There have been controversies with linking national electronic health record systems and using them for health care improvement and research in countries such as in England, Australia, and the Netherlands^1,2^. For example, Garret et al. pointed out that it may not be possible to successfully establish nationwide electronic medical records systems if such systems ignore the traditional moral orders that govern the generation, ownership, use, and responsibility for medical records^2^. Another example is the care.data program from England’s National Health Service (NHS), which adopted a scheme in which medical record data could be collected monthly and used for health care and research unless the patient specifically opted out^3^. However, care.data was shut down in 2016 because of public opposition^4,5^. Some of the many reasons for its failure were a lack of communication to the public about the project, a lack of transparency, the failure of the opt-out scheme, people’s aversion to selling their data to for-profit companies, concerns about the impact of large-scale data collection and sharing on patient privacy, the utilization of personal health data conflicting with the individual’s moral values, and a lack of social license^3,4,6^. For the purposes of the current study, “[a] social licence in the context of data-intensive health research refers to the non-tangible societal permission or approval that is granted to either public or private researchers and research organizations beyond compliance with laws and regulations”^7^.

On the other hand, the ScottisH Informatics Program (SHIP; http://www.scot-ship.ac.uk) is an example of a program recognizing the importance of public engagement and understanding. SHIP is a research platform designed for collecting, managing, distributing for research use, and analyzing 30 years’ worth of electronic patient records throughout Scotland. The SHIP system works across organizational boundaries to nationally link health and nonhealth data while protecting patient confidentiality. SHIP recognizes the importance of public trust in data handling and in addressing ethical concerns^8^. Therefore, its core projects involve public engagement activities with various objectives, such as understanding Scottish citizens’ preferences, interests, and concerns regarding sharing health data for research^9^.

Japan has also attempted to utilize health care data. In 2009, the National Database of Health Insurance Claims and Specific Health Check-Ups of Japan (NDB) was developed by the Japanese government to cover almost all health insurance schemes^10^. Most Japanese citizens are covered by employer- or community-based social insurance, so the database aimed to accumulate monthly health insurance claims data and annual specific health check-up data. Thus, the NDB is one of the most comprehensive national-level medical databases in the world and has been used as secondary data for research purposes since 2011. However, the inability to link databases related to health and long-term care was a critical difficulty in the secondary use of data and digital transformation. To realize a digital society, the Cabinet of Japan decided on the cloud-by-default principle in 2017 and issued the international *Data Free Flow with Trust* policy in 2019^11,12^. The cabinet introduced the Data Health Reform initiative in 2020 to effectively promote the use of genomic medicine, artificial intelligence (AI), personal health records (PHRs) linked to personal data, information in medical and long-term care settings, and databases^13^.

As the scale of secondary databases becomes enormous, cloud computing becomes essential for effective data management and processing. However, many ethical issues related to cloud computing (the cloud) have been pointed out in the literature. For instance, the cloud consists of complex interconnections between multiple services provided by different vendors and an equally complex network of people, blurring the boundaries of responsibility and accountability^14^. Cloud service companies collect various types of data by employing sophisticated matching algorithms in the case of an accident or other incident, which often requires highly detailed personal information to ensure accountability. However, it is difficult for users to recognize what kind of data is being recorded, and they rarely know what data will be disclosed if an incident occurs. Furthermore, users sometimes do not understand which providers store and share their data because of the complexity of cloud interconnections.

Additionally, cloud users inevitably relinquish control over their data by placing it on a provider’s server, making it difficult to know what data will be used for what purposes and leading to issues surrounding data control and ownership. Data are entrusted to cloud providers, so the employees of provider organizations can access the data at any time and from any location. Hence, data subjects’ ability and right to control their data may be limited or even violated. Even though it is important to track users’ activities on the cloud, these data can be exploited and misused if not properly protected. Therefore, Murphy and Rocchi pointed out that cloud users are the most vulnerable stakeholders in the complex web of interactions that cloud services create^15^.

To further enhance medical development, it is necessary to promote the utilization of PHRs and gain public trust and willingness to share such information. Especially for Japan, where the government has begun to push for digital transformation strongly, knowing the trust and willingness of the public is essential for risk communication. However, as far as we have found, there has been no research on what the Japanese public thinks about their PHRs, including genome data, being shared for health care research and the use of the cloud. For instance, the Study Group on Utilization of Health, Medical, and Long-Term Care Information in Japan conducted a survey to identify issues with the appropriate diffusion and development of private PHR services, but their survey was not related to the research use of PHRs^16^. Nakada et al. examined the public attitudes that focus on the secondary uses of patient records for pharmaceutical companies’ activities, and in the study, the patient records used by the research institutes were beyond their scope^17^. Asai et al. also surveyed public attitudes toward the secondary use of medical data and samples for research without specific informed consent by the focus group; however, it did not specify what entities, such as research institutes or private companies, would utilize the medical records data for research^18^. While recognizing the valuable lessons from previous studies, we found the necessity of adopting a different approach in our survey. Therefore, we carried out a survey to identify public attitudes toward cloud computing and willingness to share PHRs and genome data for health care research in Japan. In this study, we defined PHRs as medical records, genomic testing, medical check-up, smartphone health application software (app), and wearable device health data.

Based on the research results, we discuss here the current status and challenges of data sharing via the cloud and what the public deems necessary for safe data sharing in health care research. It is noteworthy that our study is also unique in regard to examining changes in people’s willingness to share data (WTSD) and PHRs for health care research, with a focus on digital health basic literacy scores (BLSs), to analyze the public’s perception of using points redeemable for goods and services as an incentive.

## Materials and methods

The main purpose of this study was to clarify the Japanese public’s attitudes and values toward sharing PHRs, including genome data, for health care research by quantifying those attitudes. Therefore, we commissioned a research company (Intage Inc., Tokyo) to recruit Japanese participants from its database (a range of everyday Japanese citizens selected based on region, gender, and age). We asked those who agreed to participate to complete the anonymous online survey, and we collected data between March 22 and 24, 2021.

In previous studies, many efforts have been made to develop genetic literacy assessment tools or methods. For instance, Erby^19^, Hooker^20^, Milo^21^, and their respective colleagues tried to create instruments assessing patients’ genetic literacy in medicine. Abrams et al. ^22^ created instruments to assess and characterize the public’s genetic literacy in the United States. Bowling^23^ developed a genetic literacy assessment instrument for undergraduate students. Sanderson et al. ^24^ developed a questionnaire to measure knowledge of whole-genome sequencing among patients and other stakeholders, such as research participants, the public, students, and health care professionals. In Japan, Ishiyama et al. ^25^ developed a quantitative scoring method to analyze the public knowledge and perception of genomic research. Subsequently, Hishiyama et al. ^26^ modified this method to examine the relationship between regulations regarding and the utilization of genetic information in medicine and research; however, PHRs in general were outside their surveys’ research scope. Although there is much to be learned from these studies, we could not find established methods for quantitatively assessing the public’s recognition and knowledge of digital health basic literacy or the relationship between the public’s perceptions of and willingness to share the PHRs included in genome data in the context of health care research. Thus, we experimentally created BLSs to examine the relationship between participants’ BLSs and their perceptions of data sharing and the use of the cloud for health care research.

Referencing previous studies, we developed a 40-question online survey to assess participants’ recognition and knowledge of six selected terms: genome, genomic medicine, cloud computing, AI, app, and wearable device (Table 1). To determine the respondents’ digital health BLSs, we asked about their level of recognition and knowledge about the terms (Table 1, Item a); for each question within that item, we assigned one point for “I have heard the term,” two points for “I understand the term’s meaning,”, and zero points for “I have never heard the term”. We then asked the respondents to indicate “right” and “wrong” definitions of the terms (Table 1, Item b). For each question within that item, we assigned one point for a correct answer and zero points otherwise. We determined scores for 12 questions and then summed the scores to produce an overall digital health BLS ranging from 0 to 18. We then divided the respondents into two groups based on the mean and median BLSs: a low-BLS group (0 ≦ BLS < 10) and a high-BLS group (10 ≦ BLS ≦ 18).

**Table 1:** Recognition and understanding of terms.

**a. Did you know the following terms prior to the survey? (*n*** **=** **5802)**	**I have heard the term**. ***n*** **(%)**	**I understand the term’s meaning**. ***n*** **(%)**	**I have never heard the term**. ***n*** **(%)**
1) Genome	3702 (63.8)	818 (14.1)	1282 (22.1)
2) Genomic medicine	2840 (48.9)	531 (9.2)	2431 (41.9)
3) Cloud computing	2899 (50.0)	2223 (38.3)	680 (11.7)
4) Artificial intelligence (AI)	2819 (48.6)	2431 (41.9)	552 (9.5)
5) Application software (App)	1886 (32.5)	3688 (63.6)	228 (3.9)
6) Wearable device	1903 (32.8)	1380 (23.8)	2519 (43.4)
**b. Is the following statement right or wrong? (*n*** **=** **5802)**	**Answered correctly** ***n*** **(%)**	**Answered incorrectly** ***n*** **(%)**	**Answered** “**I don' know**” ***n*** **(%)**
1) The genetic information of a genome exist in the DNA inside cells.	2739 (47.2)	131 (2.3)	2932 (50.5)
2) Medical treatment can be tailored to an individual’s constitution and medical condition based on personal genome information.	2847 (49.1)	116 (2.0)	2839 (48.9)
3) Cloud computing data is managed entirely by domestic servers.	2205 (38.0)	473 (8.2)	3124 (53.8)
4) Implementing artificial intelligence in the healthcare field requires training the AI with large amounts of patient data.	4191 (72.2)	156 (2.7)	1455 (25.1)
5) Apps are only activated when they are in use.	2637 (45.5)	1531 (26.4)	1634 (28.2)
6) If your wearable device shows that your heart rate, electrocardiogram, etc. are within normal limits, you don’t need to get a medical checkup.	3146 (54.2)	216 (3.7)	2440 (42.1)

We conducted descriptive statistical and binomial logistic regression analyses to explore the relationships among demographic characteristics, BLSs, and WTSD. Regarding the latter, the objective variable was a binary value for the BLSs (i.e., high BLS = 1, low BLS = 0). The explanatory variables were gender, age, educational background, annual household income, occupation, marital status, and history of outpatient visits to medical facilities in the previous year. Regarding age, we selected the value for those in their 40 s as the baseline because the majority of the respondents were in their 40 s. In terms of annual household income, we set “more than 3 million JPY to less than 5 million JPY” as the baseline for comparison because it included the median household income in Japan. Regarding educational level, we chose junior and senior high school graduates as the baseline to reflect Japan’s compulsory education system.

We used IBM® SPSS® version 27.0.1.0. for the statistical analysis. We calculated the mean, median, and standard deviation (SD) of the BLSs and then performed a *t* test or one-way analysis of variance (ANOVA) to confirm the differences.

### Ethical considerations

No ethical review was required for our study in Japan because it was outside the scope of the *Ethical Guidelines for Medical and Biological Research Involving Human Subjects* issued by the three Japanese ministries^27^. However, we followed the research ethics guidelines of the Japan Sociological Society and designed the questionnaire so that the survey participants could easily refrain from answering questions, thus reducing the burden associated with answering the questions.

## Results

We conducted an online survey to examine public attitudes toward the use of PHRs, including genome data and the cloud for health care research. The response rate was 20.4% (*n* = 5830), and Table 2 shows the participants’ demographic characteristics. The respondents who chose the “other” gender classification (*n* = 28) were excluded from the analysis because there were not enough of them to ensure statistical significance. Certain groups were asked additional questions. Questions regarding genomic data usage were addressed to direct-to-consumer (DTC) genetic testing consumers (*n* = 151, 2.6%) and those interested in consuming DTC genetic testing services (*n* = 1138, 19.6%). Similarly, we only addressed questions about accessing health data from wearable devices (e.g., smartwatches) to participants who indicated using such devices (*n* = 488, 8.4%).

**Table 2:** Relationship between sample characteristics and basic literacy scores.

Characteristics	*n* (%)	Digtal-health BLS^a^			Tendency to be in high^d^ BLS group		
		Mean	Standard deviation	*p* value	Odds ratio	95% C.I.	*p* value
Total	5802 (100.0)	9.6	4.5	----	----	----	----
Digital-health basic literacy							
Low BLS group	3461 (59.7)	6.6	3.0	----	----	----	----
High BLS group	2341 (40.3)	14.1	2.1	----	----	----	----
Gender				<0.001^b^			
Female	2897 (49.9)	8.7	4.1		1.00	----	----
Male	2905 (50.1)	10.6	4.7		1.90	1.67–2.15	<0.001*
Age Group (years old)				<0.001^c^			
20–29	886 (15.3)	9.2	4.6		0.75	0.60–0.92	0.007*
30–39	1046 (18.0)	9.0	4.5		0.85	0.71–1.01	0.063
40–49	1395 (24.0)	9.3	4.5		1.00	----	----
50–59	1265 (21.8)	10.2	4.6		1.26	1.07–1.49	0.006*
60–69	1210 (20.9)	10.3	4.3		1.30	1.09–1.55	0.004*
Household income (JPY/year)				<0.001^c^			
Less than 3 million	1421 (24.5)	8.6	4.5		0.92	0.78–1.08	0.300
3 million to less than 5 million	1598 (27.5)	9.2	4.3		1.00	----	----
5 million to less than 8 million	1596 (27.5)	10.0	4.4		1.28	1.10–1.49	0.002*
8 million to less than 20 million	1187 (20.5)	11.1	4.5		1.62	1.37–1.92	<0.001*
Educational level				<0.001^c^			
Junior or senior high school	1880 (32.4)	8.2	4.2		1.00	----	----
Occupational school or junior college	1375 (23.7)	8.9	4.1		1.38	1.18–1.62	<0.001*
University or graduate school	2547 (43.9)	11.1	4.5		2.71	2.37–3.11	<0.001*
Occupations				<0.001^c^			
Temporary worker	1143 (19.7)	8.7	4.3		0.84	0.71–1.00	0.053
Homemaker and other	1615 (27.8)	9.0	4.3		0.90	0.77–1.06	0.216
Permanent worker	2325 (40.1)	10.3	4.6		1.00	----	----
Self-employed business worker or freelance worker	475 (8.2)	10.5	4.4		1.19	0.96–1.47	0.111
Student	244 (4.2)	10.8	4.7		2.13	1.54–2.94	<0.001*
Marital status				0.157^b^			
Unmarried	2334 (40.2)	9.5	4.6		1.00	----	----
Married	3468 (59.8)	9.7	4.5		0.82	0.72–0.49	0.005*
Outpatient visits to medical facilities within the past one year				<0.001^c^			
No history of outpatient visits	2543 (43.8)	8.9	4.7		1.00	----	----
Outpatient visits within the past one year	1290 (22.2)	10.2	4.2		1.50	1.30–1.74	<0.001*
Currently receiving outpatient care at a hospital	1969 (33.9)	10.2	4.4		1.51	1.32–1.73	<0.001*
Awareness of direct-to-consumer genetic testing service				<0.001^b^			
I knew.	2373 (40.9)	12.1	3.8		----	----	----
I did not know.	3429 (59.1)	7.9	4.2		----	----	----
Interest in purchasing direct-to-consumer genetic testing services				<0.001^c^			
I have purchased the service.	151 (2.6)	11.7	4.1		----	----	----
I would like to purchase the service.	1138 (19.6)	11.5	3.8		----	----	----
I do not want to purchase	2294 (39.5)	9.4	4.6		----	----	----
I did not know about the services.	2219 (38.2)	8.8	4.5		----	----	----
Possession and type of mobile phone							
I have a smart phone.	5205 (89.7)	----	----	----	----	----	----
I have a traditional mobile phone (feature phone).	398 (6.7)	----	----	----	----	----	----
I have neither a smart phone nor a traditional mobile phone (feature phone).	120 (2.1)	----	----	----	----	----	----
I don't want to answer.	91 (1.6)	----	----	----	----	----	----

^a^Basic literacy score 0–18.

^b^Based on *t*-test comparing mean values.

^c^Based on one-way ANOVA comparing menan values.

^d^0 ≦ Low BLS < 10, 10 ≦ High BLS ≦ 18.

^e^Binomial logistic regression analysis (Low BLS = 0, High BLS = 1).

*p < 0.05.

### The public’s recognition and knowledge of terms relating to digital health and BLSs

First, we examined the participants’ recognition and knowledge of the six terms shown in Table 1. Over half of the participants indicated that they had heard of the terms or that they understood their meanings, as follows: genome 77.9%, genomic medicine 58.1%, cloud computing 88.3%, AI 90.5%, app 96.1%, and wearable device 56.6%. However, regarding the definitions of the terms, over 50% answered incorrectly or chose “I don’t know,” as follows: genome 52.8%, genomic medicine 50.9%, cloud computing 62.0%, app 54.6%, and wearable device 45.8%. For AI, the rate of incorrect/“don’t know” responses was 27.8%. Notably, 88.3% had at least heard of the cloud; however, the correct answer rate for their understanding of it was only 38.0%.

The descriptive statistics for the digital health BLSs were mean 9.6, median 10.0, and SD 4.5 for all respondents (Table 2). Statistical significance was confirmed for gender, age group, household income, education level, occupation, and history of outpatient visits (*p* < 0.001). For the demographic characteristics, those in socially disadvantaged positions tended to have lower mean BLSs; such respondents included women (M = 8.7, SD = 4.1), those with low annual household incomes (e.g., less than 3 million JPY: M = 8.6, SD = 4.5), those with less education (e.g., junior and senior high school: M = 8.2, SD = 4.2), and those with unstable occupations (e.g., temporary workers: M = 8.7, SD = 4.3; *p* < 0.001). Additionally, those who knew about DTC genetic testing services had a higher mean (M = 12.1, SD = 3.8) than those who did not (M = 7.9, SD = 4.2; *t* (5430) = 39.299, *p* < 0.001).

### Public concerns about sharing PHRs without identifiers for health care research

**Fig. 1:**
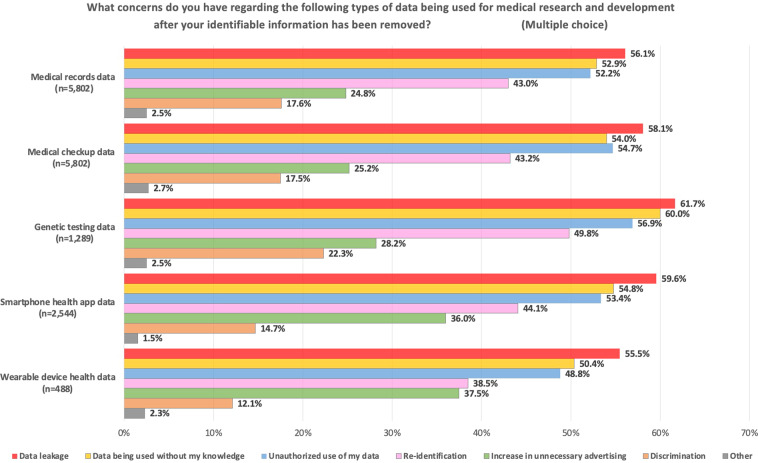
The public’s concerns about the use of PHR data without identifiers for health care research. The results of the questionnaire asking participants about their concerns regarding the use of different types of data for medical research after their identifiable information had been removed. The multiple-choice answers revealed that regardless of the data type, the respondents’ top three concerns were data leakage (≧55.5%), data being used without their knowledge (≧50.4%), and unauthorized use of their data (≧48.8%).

Figure 1 depicts the participants’ concerns about providing their medical record, medical check-up, genetic testing, smartphone health apps, and wearable-device health data without identifiers for health care research. Regardless of the data type, the respondents’ top three concerns were the same: “data leakage” (≧55.5%), “data being used without my knowledge” (hereafter “unrecognized data use”) (≧50.4%), and “unauthorized use of my data” (i.e., data used without permission; hereafter “unauthorized data use”) (≧48.8%). Especially in the case of genomic testing data, the percentages for the aforementioned three concerns and those about discrimination were the highest (data leakage 61.7%, unrecognized data use 60.0%, unauthorized data use 56.9%, and discrimination 22.3%). Notably, of the respondents, nearly 40% (≧38.5%) were concerned about possible reidentification for all types of data used for health care research despite the removal of unique identifiers to ensure anonymity.

### Relationship between the public’s views on the cloud and digital health BLSs

We asked the respondents about their views of the cloud itself. Table 3 shows the questions and results. Item a asked about their concerns regarding data-type handling via the cloud; item b asked about their perceptions of the advantages and drawbacks of using the cloud.

**Table 3:** The public’s views on cloud computing according to digital health BLSs.

**a. How do you feel about the following types of data being stored and analyzed by cloud computing? (*n*** **=** 5802)*		***n*** **(%)**	**Mean**	**Standard deviation**	***p*** value**
1) Transaction records with financial institutions	Concerned	4337 (74.8)	10.2	4.3	<0.001
	Unconcerned	632 (10.9)	10.9	4.2	
	Don't know	833 (14.4)	5.9	4.0	
2) Purchase history	Concerned	3717 (64.1)	10.0	4.3	<0.001
	Unconcerned	1258 (21.7)	11.1	4.2	
	Don't know	827 (14.3)	5.8	3.9	
3) Travel history	Concerned	3481 (60.0)	10.1	4.3	<0.001
	Unconcerned	1341 (23.1)	11.0	4.0	
	Don't know	980 (16.9)	6.1	4.0	
4) Medical record data	Concerned	3148 (54.2)	10.1	4.4	< 0.001
	Unconcerned	1603 (27.6)	10.9	4.0	
	Don't know	1051 (18.1)	6.4	4.1	
5) Genetic testing data	Concerned	3115 (53.7)	10.4	4.4	< 0.001
	Unconcerned	1248 (21.5)	11.0	4.1	
	Don't know	1439 (24.8)	6.9	4.1	
6) Medical checkup data	Concerned	2972 (51.2)	10.0	4.4	< 0.001
	Unconcerned	1831 (31.5)	10.8	4.1	
	Don't know	999 (17.2)	6.4	4.1	
7) Smartphone health app data	Concerned	2834 (48.9)	9.9	4.4	< 0.001
	Unconcerned	1872 (32.3)	10.9	4.1	
	Don't know	1096 (18.9)	6.8	4.2	
8) Wearable device health data	Concerned	2652 (45.7)	9.9	4.4	< 0.001
	Unconcerned	1717 (29.6)	11.1	4.0	
	Don't know	1433 (24.7)	7.5	4.4	
**b. Recognition of advantages and concerns about cloud computing (*n*** **=** 5802)*		***n*** **(%)**	**Mean**	**Standard deviation**	***p*** value**
Recognition of advantages about cloud computing					
1) Cloud computing is useful for backing up data in the event of a disaster.	Agree	2911 (50.2)	11.8	3.8	<0.001
	Disagree	1234 (21.3)	8.4	4.4	
	Don't know	1657 (28.6)	6.8	3.8	
2) Cloud computing is a useful way to manage large data sets.	Agree	2611 (45.0)	12.1	3.7	<0.001
	Disagree	1,418 (24.4)	8.6	4.4	
	Don't know	1773 (30.6)	6.9	3.8	
3) Cloud computing is operationally stable.	Agree	1817 (31.3)	12.1	3.8	<0.001
	Disagree	1961 (33.8)	9.5	4.5	
	Don't know	2024 (34.9)	7.6	4.0	
4) Cloud computing provides reliable security measures.	Agree	1224 (21.1)	11.5	4.0	<0.001
	Disagree	2635 (45.4)	10.3	4.4	
	Don't know	1943 (33.5)	7.6	4.2	
Concerns about cloud computing					
5) Cloud computing raises concerns about security.	Agree	3405 (58.7)	10.9	4.0	<0.001
	Disagree	1121 (19.3)	9.3	4.7	
	Don't know	1276 (22.0)	6.5	3.9	
6) Cloud computing raises concerns about the preservation of data when cloud providers take over the business of other companies.	Agree	3060 (52.7)	11.4	3.9	<0.001
	Disagree	1226 (21.1)	9.0	4.6	
	Don't know	1516 (26.1)	6.7	3.8	
7) The legislation for cloud computing is not yet firmly established.	Agree	2875 (49.6)	11.2	4.1	<0.001
	Disagree	1062 (18.3)	9.0	4.7	
	Don't know	1865 (32.1)	7.6	4.1	
8) Cloud computing raises concerns because the location of data management is not known.	Agree	2544 (43.8)	10.9	4.0	<0.001
	Disagree	1752 (30.2)	10.3	4.7	
	Don't know	1506 (26.0)	6.7	3.9	

*Five-point scale was adopted in the original questions.

**Based on one-way ANOVA comparing menan values.

Regarding item a, the respondents were more concerned about their transaction records with financial institutions (74.8%), their purchase histories (64.1%), and their travel histories (60.0%) than with their PHRs being handled via the cloud, that is, their medical record data (54.2%), genome data (53.7%), medical check-up data (51.2%), smartphone health app data (48.9%), and health data from wearable devices (45.7%). For all types of data, the mean BLS for respondents who answered that they were “unconcerned” about their data being handled via the cloud was statistically significant and higher than the mean for respondents who answered “concerned” or “I don’t know” (*p* < 0.001; Table 3, Item a).

Regarding item b on the advantages and challenges of the cloud, 50.2% of respondents considered the cloud useful for backing up data, and 45% recognized that the cloud was useful for managing large datasets. However, 58.7% of the respondents were concerned about the security of the cloud, and only 21.1% agreed that the cloud’s security measures were trustworthy. Additionally, 52.7% were concerned about data preservation following cloud provider takeovers, and 49.6% considered the legislation for cloud preservation inadequate. In addition, 43.8% were concerned about the data management location being unknown in cloud computing (Table 3, Item b).

Notably, the mean BLS for respondents who selected “agree” (≧11.47) for all advantages listed in the questions was significantly higher than the mean for respondents who selected “disagree” (≧8.37) or “I don’t know” (≧6.80; *p* < 0.001). Furthermore, the mean BLS for those who selected “agree” (≧10.93) for all concerns listed in the questions was significantly higher than the mean for respondents who selected “disagree” (≧8.99) or “I don’t know” (≧6.46; *p* < 0.001). In short, the mean BLS values for both the respondents who recognized benefits and those concerned about the cloud were significantly higher than those for the other respondents.

### Relationship between digital health BLSs and the impact of incentives for WTSD on health care research

The respondents were divided into two groups based on mean and median values: a low-BLS group (0 ≦ BLS < 10) and a high-BLS group (10 ≦ BLS ≦ 18). We then conducted a binominal logistic regression analysis. The results showed that the high-BLS group’s BLSs ranged from 11.0 to 18.0 (mean 14.1, median 14.0, and SD 2.1), whereas the low-BLS group’s BLSs ranged from 0.0 to 14.0 (mean 6.6, median 7.0, and SD 2.9; Table 2). Notably, the mean and median of the high-BLS group were approximately twice those of the low-BLS group.

Gender, age group, annual household income, education level, occupation, marital status, and history of outpatient visits were significantly associated with a tendency to be in the high-BLS group (Table 2). For instance, males had a greater tendency to be in the high-BLS group (odds ratio [OR] = 1.90, 95% confidence interval [CI] 1.67–2.15, *p* < 0.001). The respondents with “5 million JPY to less than 8 million JPY” (OR = 1.28, 95% CI 1.10–1.49, *p* = 0.002) and those “8 million JPY to less than 20 million JPY” (OR = 1.62, 95% CI 1.37–1.92, *p* < 0.001) tended to be in the high-BLS group, unlike those with “3 million JPY to less than 5 million JPY.” Regarding educational level, the OR for “occupational school or junior college” (OR = 1.38, 95% CI 1.18–1.6 2, *p* < 0.001) was larger than that for “junior or senior high school.” Notably, the OR for “university or graduate school” was more than twice (OR = 2.71, 95% CI 2.37–3.11, *p* < 0.001) that for “junior or senior high school”. Furthermore, those who had a history of outpatient visits had a statistically significant tendency to be in the high-BLS group: the values for “outpatient visit within the past year” (OR = 1.50, 95% CI 1.30–1.74, *p* < 0.001) and “currently receiving outpatient care at a hospital” (OR = 1.51, 95% CI 1.32–1.73, *p* < 0.001) were higher than those for “no history of outpatient visits”.

**Fig. 2:**
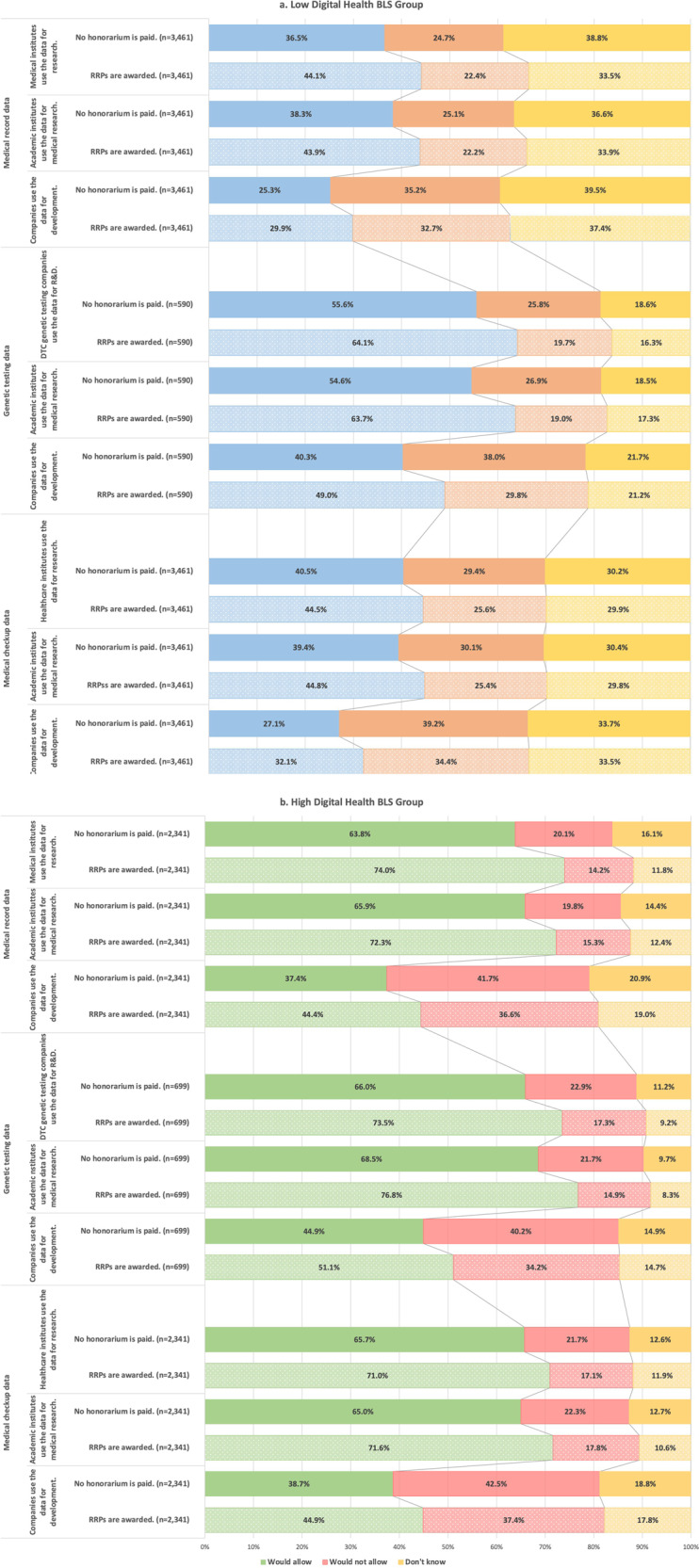
Relationship between digital health basic literacy scores (BLSs) and incentives to share health data for health care research. The results for the low and the high digital health BLS groups’ willingness to share data (WTSD) for health care research in two hypothetical situations: (1) the respondents received no honorarium and (2) the respondents received redeemable reward points (RRPs). **a** The results of the low-BLS group, **b** the results of the high-BLS group. Notably, even in the absence of an honorarium, the high-BLS group demonstrated a higher level of WTSD compared to the low-BLS group.

Although we considered five types of PHR data, including app and wearable device data, we present the results for medical record data, genomic testing data, and medical check-up data. In Japan, medical records sometimes contain medical test data, but medical check-ups do not. The respondents’ WTSD differed based on which organization used their data. In addition, their WTSD differed according to whether they were in the high- or low-BLS group. Figure 2 shows the results for each group’s willingness to share their medical record, genetic testing, and medical check-up data for health care research in two hypothetical situations: (1) the respondents received no honorarium and (2) the respondents received redeemable reward points (RRPs). The high-BLS group had a greater WTSD than the low-BLS group, even when honorariums were not awarded. Except for the case of private companies, the percentage of respondents who indicated their WTSD without honorariums in the high-BLS group was over 63.8%, and the percentage exceeded 70% when RRPs were awarded for all three types of data. Even in the low-BLS group, the willingness to share genomic testing data exceeded 50% without honorariums.

When RRPs were awarded, an increase in the percentage of WTSD was observed for all types of data in both BLS groups. The supplementary material includes the results without differentiating between high and low BLS; it shows that regardless of the data type, the number of respondents who were WTSD increased with awarded RRPs, but the increase only reached 8.7%, whereas in the low-BLS group, the highest increase was observed at 9.1% when there were RRPs, which was the case for sharing genomic testing data with a research institute. In the high-BLS group, the largest increase was 10.2% for the use of medical record data by the acquiring institution; however, in other cases, the increase was less than 8.5%.

Overall, in both the high- and low-BLS groups, the WTSD with private companies was lower than in cases where data were shared with acquiring institutions or research institutes, regardless of whether RRPs were awarded.

Finally, the number of respondents who answered “I don’t know” regarding whether they would share data with organizations for research without honoraria was higher in the low-BLS group, especially in the case of medical record data, which was at approximately 40%, regardless of the organization. However, in the high-BLS group, the maximum percentage was 20.9%.

## Discussion

Although the use of BLSs was a pilot method for digital health basic literacy assessment, it helped in understanding the attitudes of the general Japanese public. The BLSs identified a high level of recognition of certain terms, including genome and the cloud, but these terms were not well understood. Therefore, researchers need to be careful about the usage of technical terms when seeking informed consent or creating opt-out forms because even highly recognized terms may not be actually understood. These results also suggest that some support is needed to improve the general public’s digital health literacy, especially for those with low BLSs, in Japan.

However, the public’s perceptions of the advantages and challenges of the cloud indicated that those with a high mean BLS were both aware of the advantages of the cloud and simultaneously more concerned about it than others were. This shows that improving BLSs or knowledge does not necessarily alleviate concerns.

The survey results also highlighted that the primary concerns about using PHRs for health care research were leakage, unauthorized data use, and unrecognized use of data. The participants might have been more concerned about breaches of confidentiality or the potential abuse or exploitation of their data. Therefore, it is important to build trust among all stakeholders, to provide opportunities for research participants and the public to learn about digital health and data-intensive health care research and be empowered. This is particularly pertinent to Japan’s data health reforms.

The issues of data leakage, unauthorized data use, and unrecognized data use overlapped with cloud-related issues. For the research participants, the concerning factors with data sharing for medical research and data deposition in the cloud may be the difficulty or inability of controlling PHR data once it is shared. This can be more prominent in regard to the secondary use of data for future health care research. Additionally, researchers’ collaborations with for-profit companies could raise concerns regarding the ambiguity of the companies’ roles, the commercialization of research, and the use of data beyond the terms of the partnerships^28^. Therefore, despite the substantial benefits for researchers in regard to using the cloud, the benefits are less evident to those research participants who only provide data.

Concerning the relationship between incentives and WTSD, we found that WTSD varied significantly according to the BLS. The high-BLS group was more positive about providing data for health care research than the low-BLS group. Interestingly, regardless of the BLS group, the respondents who were interested in DTC genetic test services tended to be more positive about sharing their genomic testing data than other types of data for health care research. Conversely, incentive-induced changes in WTSD were mostly less than 10%, implying that simply announcing incentives would most likely not lead to a significant increase in the number of registrants. Some previous studies have shown that financial incentives have a low likelihood of inducing research participants to enroll in clinical trials^29,30^. This may also be the case for incentives and WTSD in data-intensive health care research. Our research has indicated that there could be a correlation between high and low BLS and WTSD, but further research is necessary.

Additionally, we would like to emphasize that in cloud-based genomic research, research participants are even more vulnerable stakeholders than cloud users, even though Murphy and Rocchi identify the latter as the most vulnerable. Under Japanese regulations, genome data may constitute personal information even if identifiers are removed. Therefore, certain genome data pertaining to research participants will be stored by researchers as research participants’ personal information in the cloud. In this case, research participants lose a substantial amount of control over their data because they do not have any means of directly controlling it; they must rely on researchers and cloud vendors.

It must be noted that both cloud vendors and researchers, as data stewards, are responsible for keeping activity logs and personal information regarding the use of data obtained from research participants. In this sense, there is an existing structure in cloud-based research that requires the researcher to provide data to the cloud vendor. In other words, a two-tiered structural vulnerability arises in certain types of genomic research using the cloud with respect to the two parties whose personal information is registered in the cloud, and both researchers and research participants are vulnerable consequences from the data platform industry, which has more power than any one nation in the data business.

One of the key factors for successful genomic research using the cloud may be overcoming this vulnerability to diminish public concern and help build trust in research. Some scholars, including the European Data Protection Supervisor, have introduced the notion of the “prosumer” as one of the pillars of new digital ethics to overcome concerns about new technologies such as the cloud and empower users of such technologies^15^. A prosumer is a consumer and producer simultaneously, as posited by Alvin Toffler; however, some scholars have defined the term prosumer in different ways^31,32^. For instance, Prahalad and Ramaswamy proposed “cocreation” activities that include dialog, access, risk/benefits, and transparency as the basis for interaction^33^. We agree that “cocreation” would be a fitting key value in health care research because data subjects are subjects, not objects. Although the data stewardship of researchers is often espoused in health care research, it has been pointed out that the role of stewardship can be ambiguous for patients and the general public due to the complexity of cloud utilization in health research^28^. Therefore, we argue that it is vital to consider patients and the public as “value cocreators” in data-intensive health care research. In this context, patient and public involvement/engagement would help build trust among cloud providers, researchers, and the public, thus overcoming this vulnerability.

## Limitations

The present study has several limitations. The survey did not directly ask about attitudes regarding the use of cloud computing in the context of health care research. Additionally, we did not set a specific number of redeemable points. Thus, each participant may have perceived “points” differently. Moreover, because this survey was conducted with Japanese participants, further research is needed to determine whether the same conclusions can be drawn for non-Japanese participants. Finally, a separate ethical discussion is needed regarding exchanging private data for rewards.

### Supplementary information

Supplement
