# Supplementary material for: Public attitudes toward cloud computing and willingness to share personal health records (PHRs) and genome data for health care research in Japan
Source: Hum Genome Var. 2023 Mar 30;10:11. doi: 10.1038/s41439-023-00240-1 (PMC10060394; doi:10.1038/s41439-023-00240-1)

Supplement. Preference changes regarding sharing PHRs with a research institute or company because of RRPs


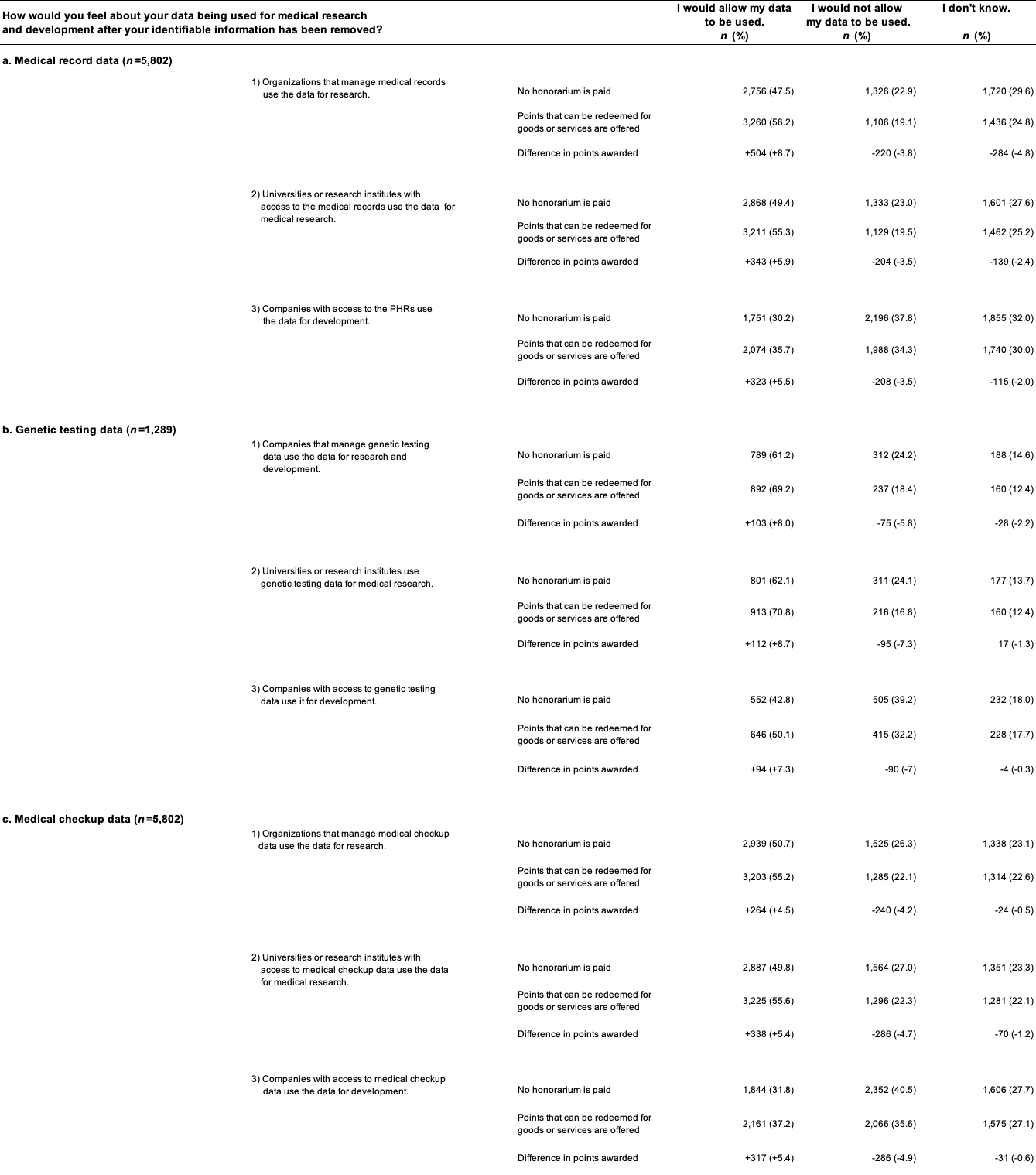

Supplement: Supplementary file 1 — Supplement [file 41439_2023_240_MOESM1_ESM.docx]
